# Management of Unruptured Small Multiple Intracranial Aneurysms in China: A Comparative Effectiveness Analysis Based on Real-World Data

**DOI:** 10.3389/fneur.2021.736127

**Published:** 2022-01-27

**Authors:** Jigang Chen, Xin Tong, Xin Feng, Fei Peng, Hao Niu, Mingyang Han, Lang Liu, Yuanli Zhao, Daming Wang, Yuesong Pan, Aihua Liu

**Affiliations:** ^1^Beijing Neurosurgical Institute, Capital Medical University, Beijing, China; ^2^Department of Interventional Neuroradiology, Beijing Tiantan Hospital, Capital Medical University, Beijing, China; ^3^Department of Neurosurgery, National Center of Gerontology, Beijing Hospital, Beijing, China; ^4^Department of Neurosurgery, The Third Xiangya Hospital, Central South University, Changsha, China; ^5^Department of Neurosurgery, Peking University International Hospital, Peking University, Beijing, China; ^6^Department of Neurology, Beijing Tiantan Hospital, Capital Medical University, Beijing, China; ^7^China National Clinical Research Centre for Neurological Diseases, Beijing, China

**Keywords:** multiple intracranial aneurysms, small aneurysm, comparative effectiveness analysis, real-world data, endovascular therapy

## Abstract

**Background:**

Unruptured small aneurysms with a size of <7 mm were often followed conservatively. However, it is unknown whether unruptured small multiple intracranial aneurysms (MIAs) are better to be prophylactically treated or conservatively followed.

**Objective:**

We aim to compare the effectiveness of different strategies regarding their management.

**Methods:**

A decision-analytic Markov model was built over a entire life cycle. The compared strategies include natural history, treat one aneurysm, treat both aneurysms, annual follow-up, biennial follow-up, and follow-up every 5 years. The inputs for the model were obtained from real-world data and related medical literature. Outcomes were measured in terms of quality-adjusted life-years (QALYs).

**Results:**

Treat both aneurysms had the highest effectiveness of 15.36 QALYs and treat one aneurysm had the second-highest effectiveness of 15.11 QALYs. Probabilistic sensitivity analysis with 10,000 iterations showed that treat both aneurysms and treat one aneurysm were optimal in 67.28 and 17.91% of all cases, respectively. One-way and two-way sensitivity analyses showed that the result was sensitive to the proportion of moderate to severe disability after treating two aneurysms, mortality after treating two aneurysms, proportion of moderate to severe disability after treating one aneurysm, and rupture rate of small growing aneurysm. Either treat both aneurysms or treat one aneurysm would be the optimal strategy under most of the circumstances with the variations of these parameters.

**Conclusion:**

For patients with small unruptured MIAs, prophylactic coiling was superior to conservative management and at least one aneurysm should be treated.

## Introduction

Intracranial aneurysms are common among healthy adults and affect approximately 3 to 7% of them (1, 2). Multiple intracranial aneurysms (MIAs) are defined as those harboring two or more aneurysms in one patient. The reported rate of MIAs among aneurysm carriers ranges between 2 and 44.9% (3). Endovascular coiling has gained wide popularity for the treatment of aneurysms during the last two decades (4). Nowadays, patients with MIAs often receive endovascular treatment because of safety and efficiency (5–9).

Patients with aneurysms are often at risk of rupture, which might lead to a devastating subarachnoid hemorrhage (SAH) and subsequent unpleasant outcomes. A large proportion of aneurysms are small with a size of <7 mm, and managing small unruptured aneurysms is one of the most controversial topics in neurosurgical medicine (10). The second International Study of Unruptured Intracranial Aneurysms has demonstrated that the risk of rupture from small aneurysms is extremely low (11). Treatment of these aneurysms brings a greater risk of unpleasant outcomes than the natural hisotry. This has led to a more conservative management approach (12). However, since a large group of patients carries small aneurysms, a significant number of SAH is actually from them. Therefore, considerable uncertainty remains regarding their management.

For small unruptured aneurysms that were managed conservatively, follow-up at regular intervals with computed tomography angiography or magnetic resonance angiography (MRA) was recommended to assess possible changes in size, because growing aneurysms are prone to rupture (12, 13). However, there is no clear consensus on the optimal management of small MIAs nowadays, since they are more likely to grow and rupture than the single ones (3, 14). Moreover, a significantly higher rate of unfavorable outcomes for the endovascular treatment of unruptured MIAs than treatment of the single aneurysm was reported (5). It is unknown whether unruptured small MIAs are better to beprophylactic treated or what the appropriate frequency and duration of follow-ups are if conservative management was performed.

In this study, we performed a comparative effectiveness analysis to evaluate six different strategies in the management of small unruptured MIAs. To make our model more simplified, we assumed that all the patients carried only two aneurysms. All the treatments were performed by endovascular coiling, and all the follow-ups were performed by MRA. The evaluated strategies included natural history, treat one aneurysm, treat both aneurysms, annual follow-up, biennial follow-up, and follow-up every 5 years.

## Materials and Methods

### Real-World Data Collection

This collection was retrospectively collected from three tertiary hospitals in Beijing. Informed consent for each patient was waived because of study design. A total of 1,334 patients who were admitted because of MIAs from January 2014 to August 2020 were included in our MIA database. The exclusion criteria were (1) patients who received open surgery, (2) traumatic, fusiform, and blood blister-like aneurysms, and (3) patients with history of other major diseases such as severe ischemic stroke, tumor, uremia, and heart failure. The collected information includes demographic characteristics, aneurysm size, aneurysm location, treatment modalities, costs, clinical outcomes, etc. Patients who had two small unruptured aneurysms were identified from this database for later analysis.

### Model Structure

We built a decision-analytic Markov model over a life span using TreeAge Pro Suite 2020 (TreeAge Software Inc.). According to our database, the average age of patients harboring MIAs was 56.8 years old. Therefore, the model starts with a 57-year-old patient with two unruptured small aneurysms. The length of one Markov cycle was 1 year, and this model would not stop until all the patients died or reached 99 years old. Nine different health states were introduced in this model, namely, well with MIAs, well with growing MIAs, well with a single growing aneurysm, well with one treated aneurysm, well with both treated aneurysms, SAH, mild disability, moderate to severe disability, and death. The branch of “Natural history” in the model in provided in Supplementary Material, and the whole model is available upon request.

For the “natural history”, all the MIAs carried an annual risk of SAH because of rupture. After rupture, all the patients with SAH would have endovascular coiling and both of the two aneurysms were assumed to be coiled. After coiling, they would have full recovery [modified Rankin scale (mRS) score of 0–1], permanent mild disability (mRS score 2), permanent moderate to severe disability (mRS score of 3–5), or die (mRS score of 6). We assumed that only those with full recovery would have annual MRA follow-up in the subsequent years due to *de novo* aneurysm formation that needs a second treatment.

For “follow-up”, MIAs would be followed annually, biennially, or every 5 years to assess the possible growth in aneurysmal size because the growing aneurysm is more likely to rupture. If size change was observed, the growing aneurysm was assumed to be coiled directly, with the non-growing one left untreated. After the treatment, patients would have a full recovery, permanent mild disability, permanent moderate to severe disability, or die. Fully recovered patients would have an annual follow-up for possible *de novo* aneurysm formation. A rupture would also occur in non-growing aneurysms and could not be prevented by imaging screening. The outcomes of treating ruptured aneurysms were the same as those of the “natural history”.

For “treat one aneurysm”, only one aneurysm was assumed to receive prophylactic coiling. The treatment outcomes were similar to those of “follow-up”. An annual follow-up would be performed among the fully-recovered patients for possible growth of the untreated one or *de novo* aneurysm formation. The untreated one also carries an annual risk of rupturing.

For “treat both aneurysms”, both aneurysms were assumed to be coiled prophylactically. The patients would also have the four aforementioned outcomes. Treated patients would have an annual follow-up for possible *de novo* aneurysm formation.

### Clinical Parameters

We retrieved all the clinical parameters from our cohort or recently published large cohort studies or meta-analysis studies whenever available. The annual growth rate (2.6%) and annual rupture rate (0.5%) of small non-growing aneurysm were obtained from a recent meta-analysis by Malhotra et al. (15). The annual rupture rate (6.3%) of small growing aneurysm was retrieved from an observational study and systematic review by Gondar et al. (16). The risk ratio of growing (3.47) and rupturing (2.08) in MIAs compared with a single aneurysm was from the meta-analysis performed by Ramazan et al. (3). The rate of *de novo* aneurysm formation was estimated to be 0.003, which was reported in a recent meta-analysis (17). The risk ratio of *de novo* aneurysm formation in patients with MIAs compared with a single aneurysm was 3.92 (3). The outcomes of endovascular treatment for unruptured MIAs were obtained from our cohort. The outcomes of treating aneurysmal SAH were estimated from a meta-analysis and the International Subarachnoid Aneurysm Trial (4, 18), in which a mortality rate of 35%, a mild disability rate of 15%, and a moderate to severe disability rate of 9% were used in our study. The age-specific mortality rates were obtained from the most recent published census of China and were adjusted by the aneurysmal SAH cause of death (19, 20). Disabled patients are at higher risk of death. The mortality rate for mildly disabled patients was adjusted by 2.02-fold, and for severely disabled it was adjusted by 4.46-fold (21).

### Utilities

Each of the health states was assigned with health-related quality of life value (utility score). Quality-adjusted life-years (QALYs) were calculated to determine health outcomes by multiplying the length of patient-years within a particular health state by the corresponding utility score. The utility scores of different health states were obtained from a previous cost-effective analysis of the preventive treatment of unruptured aneurysms (22). The coiling procedure was assumed to cause a temporary 5% disutility (23).

All the utilities were discounted by 3% annually (24). The input variables including clinical parameters and utilities are listed in Table 1.

**Table 1:** Input parameters of the decision analytic model.

**Variable**	**Mean**	**Range**	**Distribution**	**Sources**
**Clinical parameters**				
Growth rate of small aneurysm	0.026	0.017–0.04	Beta SD: 0.004	(15)
Rupture rate of small nongrowing aneurysm	0.005	0.003–0.009	Beta SD: 0.001	(15)
Rupture rate of small growing aneurysm	0.063	0.01–0.22	Beta SD: 0.035	(16)
Risk ratio of growing in MIAs compared with single aneurysm	3.47	1.87–6.45	Lognormal SD: 1.15	(3)
Risk ratio of rupturing in MIAs compared with single aneurysm	2.08	1.46–2.96	Lognormal SD: 0.25	(3)
Rate of *de novo* aneurysm formation in patients with single aneurysm	0.003	0.002–0.004	Beta SD: 0.0004	(17)
Risk ratio of *de novo* aneurysm formation in patients with MIAs compared with single aneurysm	3.92	1.95–7.87	Lognormal SD: 0.99	(3)
Proportion of mild disability after treating one aneurysm	0.016	0–0.037	Beta SD: 0.011	MIAs database
Proportion of moderate to severe disability after treating one aneurysm	0.047	0.01–0.083	Beta SD: 0.019	MIAs database
Mortality after treating one aneurysm	0	0–0.005	Beta SD: 0.001	MIAs database
Proportion of mild disability after treating two aneurysms	0.032	0–0.067	Beta SD: 0.018	MIAs database
Proportion of moderate to severe disability after treating two aneurysms	0.053	0.008–0.098	Beta SD: 0.023	MIAs database
Mortality after treating two aneurysms	0.011	0–0.031	Beta SD: 0.01	MIAs database
Proportion of mild disability after aneurysmal SAH	0.15	0.13–0.17	Beta SD: 0.007	(4, 18)
Proportion of moderate to severe disability after aneurysmal SAH	0.09	0.07–0.11	Beta SD: 0.007	(4, 18)
Mortality after aneurysmal SAH	0.35	025–0.45	Beta SD: 0.033	(4, 18)
Risk ratio of death in mild disability compared with general population	2.02	1.7–2.4	Lognormal SD: 0.109	(21)
Risk ratio of death in moderate to severe disability compared with general population	4.46	4.05–4.91	Lognormal SD: 0.128	(21)
**Utility**				
Full recovery	1			
Mild disability	0.72	0.65–0.80	Triangle	(22)
Moderate to severe disability	0.41	0.25–0.65	Triangle	(22)
SAH	0.64	0.52–0.71	Triangle	(22)
Coiling procedure	5% disutility			(23)

*MIAs, multiple intracranial aneurysms; SD, standard deviation; SAH, subarachnoid hemorrhage*.

### Validation

Model structure, data source, formula, and results were reviewed by all the authors. Internal validation was performed using the TreeAge Pro software. External validation was not available, since there were no similar published studies.

### Statistical Analysis

A base case calculation was performed using the mean value of each parameter. Probabilistic sensitivity analysis (PSA) with Monte Carlo simulation was conducted with 10,000 iterations, modeling 10,000 patients. All the parameters were assigned a distribution and varied simultaneously according to their distributions in the PSA. In addition, one-way and two-way sensitivity analyses were carried out to account for the uncertainty of specific parameters on the model outcome.

## Results

### Real-World Data

A total of 224 patients with two small unruptured aneurysms who received endovascular treatment were included in our MIA database. Mean age was 55.98 ± 9.99 years old. Among them, 129 had one aneurysm treated, and 95 patients had both two aneurysms treated. The average time between discharge and last follow-up was 31.24 ± 22.93 months. There were no differences in age, gender, aneurysm location, aneurysm size, and follow-up time between these two groups (Table 2). For patients who had both two aneurysms treated, all of them received one-stage treatment. The clinical outcomes of the two groups are presented in Table 1.

**Table 2:** Comparison among patients who had one aneurysm treated and both aneurysms treated.

**Variables**	**Treat one aneurysm (*n* = 129)**	**Treat both aneurysms (*n* = 95)**	***P*-value**
Age (years), mean (SD)	56.67 ± 9.72	55.04 ± 10.29	0.087
Female, *n* (%)	84 (65.1)	63 (66.3)	0.852
Hypertension, *n* (%)	63 (48.8)	46 (48.4)	0.951
Hyperglycemia, *n* (%)	18 (14.0)	15 (15.8)	0.702
Hyperlipidemia, *n* (%)	25 (19.4)	25 (26.3)	0.218
Coronary heart disease, *n* (%)	12 (9.3)	14 (14.7)	0.210
History of stroke, *n* (%)	18 (14.0)	17 (17.9)	0.422
Smoking, *n* (%)	32 (24.8)	15 (15.8)	0.101
Alcohol, *n* (%)	17 (13.2)	10 (10.5)	0.547
Aneurysm location, *n* (%)			0.433
Anterior cerebral artery	5 (1.9)	4 (2.1)	
Anterior communicating artery	19 (7.4)	10 (5.3)	
Internal carotid artery	194 (75.2)	159 (83.7)	
Middle cerebral artery	18 (7.0)	7 (3.7)	
Posterior cerebral artery	2 (0.8)	2 (1.1)	
Basilar artery	11 (4.3)	6 (3.2)	
Vertebral artery	5 (1.9)	2 (1.1)	
Posterior inferior cerebellar artery	4 (1.6)	0	
Irregular aneurysm shape, *n* (%)	89 (34.5)	58 (30.5)	0.376
Aneurysm size (mm), mean (SD)	4.14 ± 1.40	4.35 ± 1.25	0.097
Follow-up times (months), mean (SD)	29.52 ± 22.96	33.56 ± 22.75	0.065

*SD, standard deviation*.

### Base Case Calculation

According to the results, prophylactic treatment or follow-up would increase effectiveness. Follow-up with a shorter period of interval resulted in higher effectiveness. Treat both aneurysms was the best strategy with the highest effectiveness of 15.37 QALYs, and treat one aneurysm had the second highest effectiveness of 15.11 QALYs. Natural history was the least favorable option, which gained the lowest effectiveness of 14.31 QALYs.

### Probabilistic Sensitivity Analysis

In the PSA, we performed 10,000 iterations to simulate a cohort of 10,000 patients. When compared with treat one aneurysm (the strategy with the second highest effectiveness), treat both aneurysms was more favorable in 72.81% of iterations. This result was stable after 10 repeated analyses, indicating that these iterations were sufficient to achieve a reliable outcome.

### One-Way and Two-Way Sensitivity Analyses

**Figure 1:**
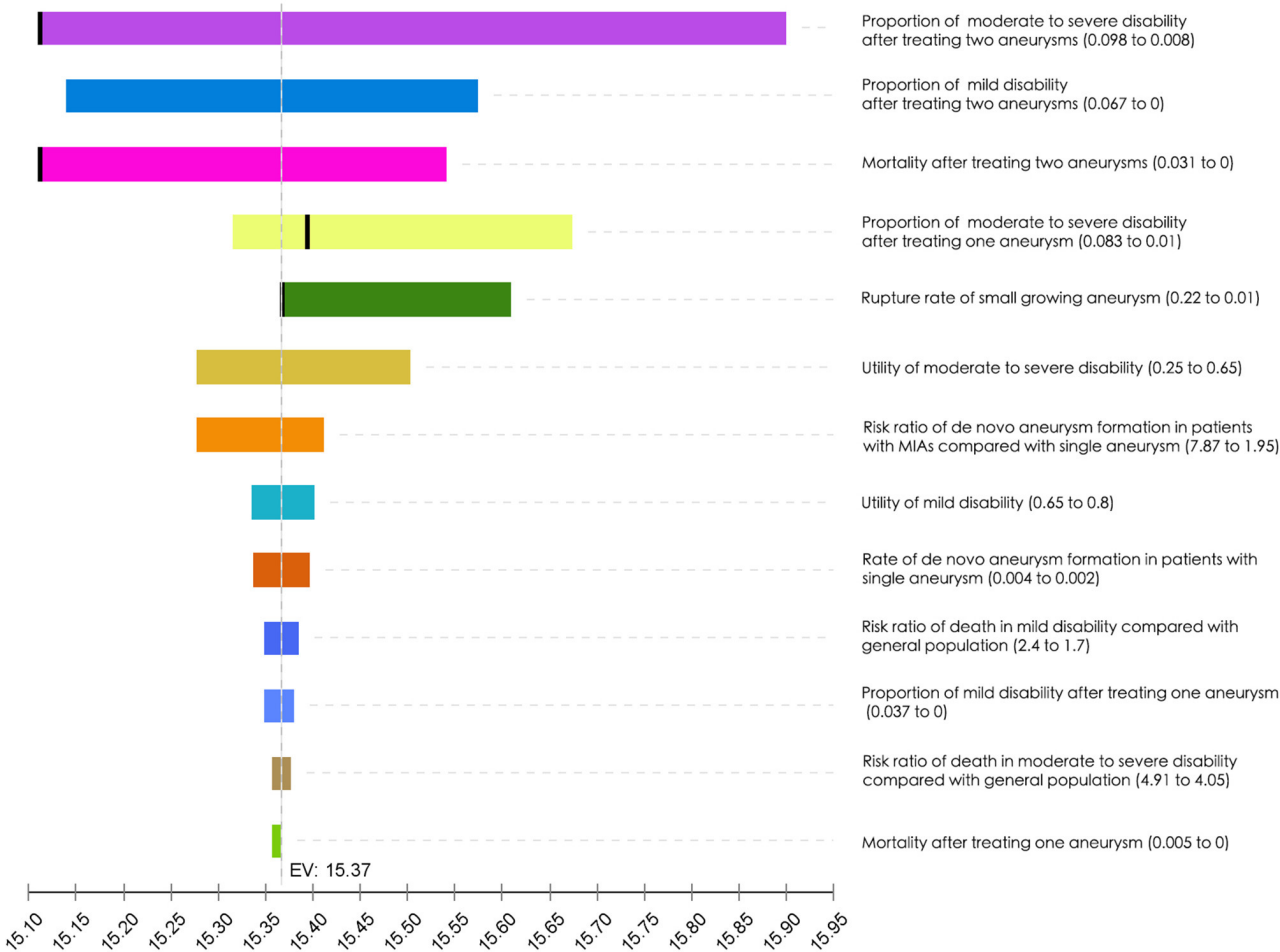
Tornado diagram. The effect of variation of each parameter on the expected value is presented on each bar. The dark line within the bar represents the alteration of the optimal strategy. EV, expected value.

One-way sensitivity analyses were performed. The results were presented in the tornado diagram, which was a set of one-way sensitivity analyses brought together in a single graph (Figure 1). According to the results, the optimal strategy was sensitive to four parameters, namely, proportion of moderate to severe disability after treating two aneurysms, mortality after treating two aneurysms, proportion of moderate to severe disability after treating one aneurysm, and rupture rate of small growing aneurysm.

**Figure 2:**
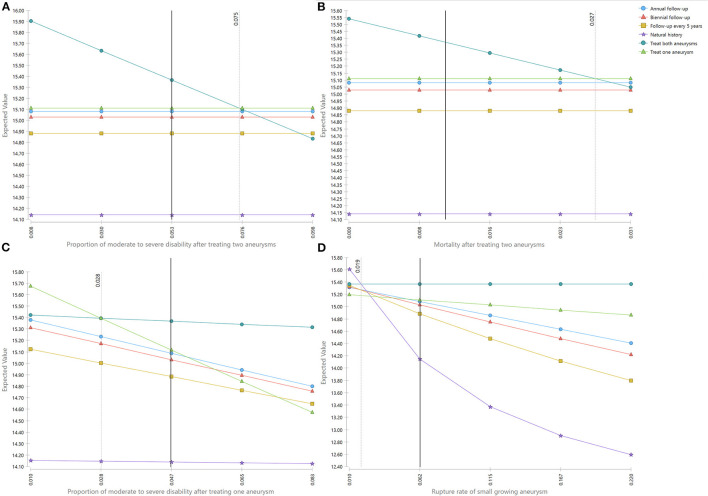
**(A–D)** One-way sensitivity analyses. The light-dark line represents the threshold. The deep-dark line represents the base case value.

When the proportion of moderate to severe disability after treating two aneurysms was <0.075, treat both aneurysms was the best strategy. When the rate was above 0.075, treat one aneurysm was more favored (Figure 2A). Similarly. When the mortality after treating two aneurysms was < 0.027, treat two aneurysms was the best option; and if this rate was higher than 0.027, treat one aneurysm turned to be the best one (Figure 2B). When the proportion of moderate to severe disability after treating one aneurysm was above 0.028, treat both aneurysms was the most favorable option. The treatment of one aneurysm would be the superior one if this proportion was < 0.028 (Figure 2C). For the rupture rate of small growing aneurysm, treat both aneurysms was the best strategy if the value was above 0.019 (Figure 2D).

**Figure 3:**
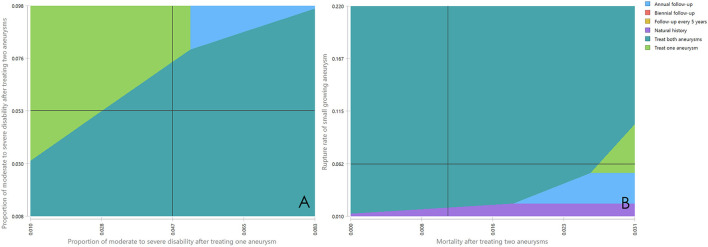
**(A,B)** Two-way sensitivity analyses. The dark line represents the base case value. The different color areas represent different optimal strategies.

To account for the uncertainty of the proportion of moderate to severe disability after treating one aneurysm and after treating both aneurysms together on the outcome, we put these two parameters in the two-way sensitivity analysis. The results showed either treat both aneurysms or treat one aneurysm would be the best option under most circumstances (Figure 3A). We also performed a two-way sensitivity analysis on the mortality after treating two aneurysms and rupture rate of small growing aneurysm, and treat both aneurysms is the best strategy by large chance (Figure 3B).

## Discussion

No specific guidelines exist regarding the management of unruptured small UIAs. In this study, we performed a comparative effectiveness analysis based on real-world data to investigate which strategy would benefit patients the most. According to the base case calculation, treat both aneurysms resulted in highest effectiveness, and treat one aneurysm gained second highest effectiveness from a lifetime horizon, meaning that patients having their aneurysms treated would have a prolonged life expectancy or improved life quality. Sensitivity analyses were performed to determine whether treat both aneurysms was better than treat one aneurysm. However, the PSA proved that treat both aneurysms is more superior to treat one aneurysm only in 72.81% of the cases. One-way sensitivity and two-way sensitivity analyses also indicated that the most favorable strategy would change between treat one aneurysm and treat both aneurysms with variations of treatment outcomes and the rupture rate of small growing aneurysm. Notwithstanding, our results together suggested that prophylactic coiling for patients with small unruptured UIAs was better than natural history or imaging follow-up.

In the MIA database, there were 89 patients with SAH due to ruptured small MIAs, and all of them received endovascular coiling. Last time follow-up showed that 80 (89.89%) of the patients had favorable outcomes (mRS 0–2), and that the mortality rate for them was 2.2%. However, we did not use these outcomes in our model, because they might not reflect the real outcomes of aneurysmal SAH. This is because a significant portion of patients with SAH would die before reaching a hospital and the actual number is difficult to estimate in China. In addition, the MIA database was created based on the clinical information from three tertiary hospitals in Beijing, and most of the patients with SAH were transferred from other areas and cities. Patients who were predicted to have unfavorable outcomes would be treated at the local hospitals and not be transferred. Only those with mild symptoms would have a chance to be treated in our centers.

According to our results, the optimal strategy is sensitive to the treatment outcomes including the proportion of moderate to severe disability after treating two aneurysms, mortality after treating two aneurysms, and proportion of moderate to severe disability after treating one aneurysm. Several studies have investigated the safety and efficiency of endovascular treatment of MIAs. However, studies reporting the outcomes of coiling unruptured MIAs are limited. Jeon et al. investigated the coiling of all aneurysms among 132 patients with unruptured MIAs, and only three (2.3%) had unfavorable outcomes (mRS score of 3–6) at discharge (7). In another study, 27 patients with unruptured MIAs underwent endovascular treatment for all aneurysms, and three (11.1%) patients died because of the treatment. The proportion of unfavorable outcomes for unruptured MIAs was not reported in this study (5). The high rate of mortality in this study came from the fact that the included cases consisted of the most complicated and complex aneurysms that were difficult to treat. A small sample size might be another important factor contributing to this high mortality rate. In our study, the unfavorable outcomes for coiling one aneurysm were 4.7% and for coiling both aneurysms was 6.4%. Our study included only small aneurysms with a size of <7 mm. Treating small aneurysms, especially tiny ones with a size of <3 mm, was sometimes particularly challenging, with high rates of complications and unfavorable outcomes (25). Therefore, it is understandable that our cohort resulted in a higher unfavorable rate than that of Jeon et al.

China has the largest population in the world, and the demand for a data-driven and evidence-supporting healthcare system has increased significantly for policymakers in China (26). As a matter of fact, comparative effectiveness or cost-effectiveness research studies have been advocated by a number of health policy reforms (27), and there are fast-growing numbers of published studies over the last two decades in China (28). Even though our study could not determine which strategy was best for the management of small unruptured MIAs, we proved that at least one aneurysm should be treated. Several aneurysm characteristics, such as size, shape, and location, were related to rupture risk (11). We suggested that for patients with small unruptured MIAs, at least the aneurysm with a higher risk of rupturing should be treated.

## Limitations

This study has several limitations. First, the real-world data were retrospectively collected, and it tends to be less reliable than the prospectively conducted studies. Second, the patients included in our study were all collected from three tertiary hospitals in Beijing; thus, our findings might not be applicable to the whole of China. Data from other regions or provinces are needed to reflect a national perspective. However, as far as we know, our database included the largest number of patients with MIAs in China, and the sensitivity analyses have accounted for the differences. Third, we performed MRA as the screening modality for aneurysms and assumed each aneurysm growth could be detected by MRA. However, are some concerns about the sensitivity and specificity of MRA for the detection of aneurysms, especially for the small ones (29). Actually, there is no published literature on the accuracy of detecting aneurysm growth (13), and the definition of growth is different among different studies. Computed tomographic angiography would have a higher spatial resolution, but it is not ideal for long-term imaging follow-up because of radiation concerns. Lastly, we do not consider the effect of complications or retreatment on the effectiveness of different management strategies. However, this is not unprecedented, and it would not affect our results to a large extent because of its low incidence (22, 30).

## Conclusions

The comparative effectiveness analysis based on real-world data suggests that for patients with small unruptured MIAs, prophylactic coiling was superior to conservative management, and that at least one aneurysm should be treated.

## Data Availability Statement

The raw data supporting the conclusions of this article will be made available by the authors, without undue reservation.

## Ethics Statement

Ethical review and approval was not required for the study on human participants in accordance with the local legislation and institutional requirements. Written informed consent for participation was not required for this study in accordance with the national legislation and the institutional requirements.

## Author Contributions

JC and XT were responsible for the design of the study. JC and AL built the model and conducted the statistical analysis. XT, XF, FP, and HN collected the real-world data. MH and LL prepared the manuscript. DW, YZ, and YP verified the data. All the authors reviewed the structure of the model, data source, formula, and results.

## Funding

This work was supported by Beijing Science and Technology Planning Project (No. Z181100009618035).

## Conflict of Interest

The authors declare that the research was conducted in the absence of any commercial or financial relationships that could be construed as a potential conflict of interest.

## Publisher's Note

All claims expressed in this article are solely those of the authors and do not necessarily represent those of their affiliated organizations, or those of the publisher, the editors and the reviewers. Any product that may be evaluated in this article, or claim that may be made by its manufacturer, is not guaranteed or endorsed by the publisher.
