# Supplementary figures and images for: Management of Unruptured Small Multiple Intracranial Aneurysms in China: A Comparative Effectiveness Analysis Based on Real-World Data
Source: Front Neurol. 2022 Jan 27;12:736127. doi: 10.3389/fneur.2021.736127 (PMC8830354; doi:10.3389/fneur.2021.736127)

eFigure: “Natural history” in the decision-analytic Markov model.

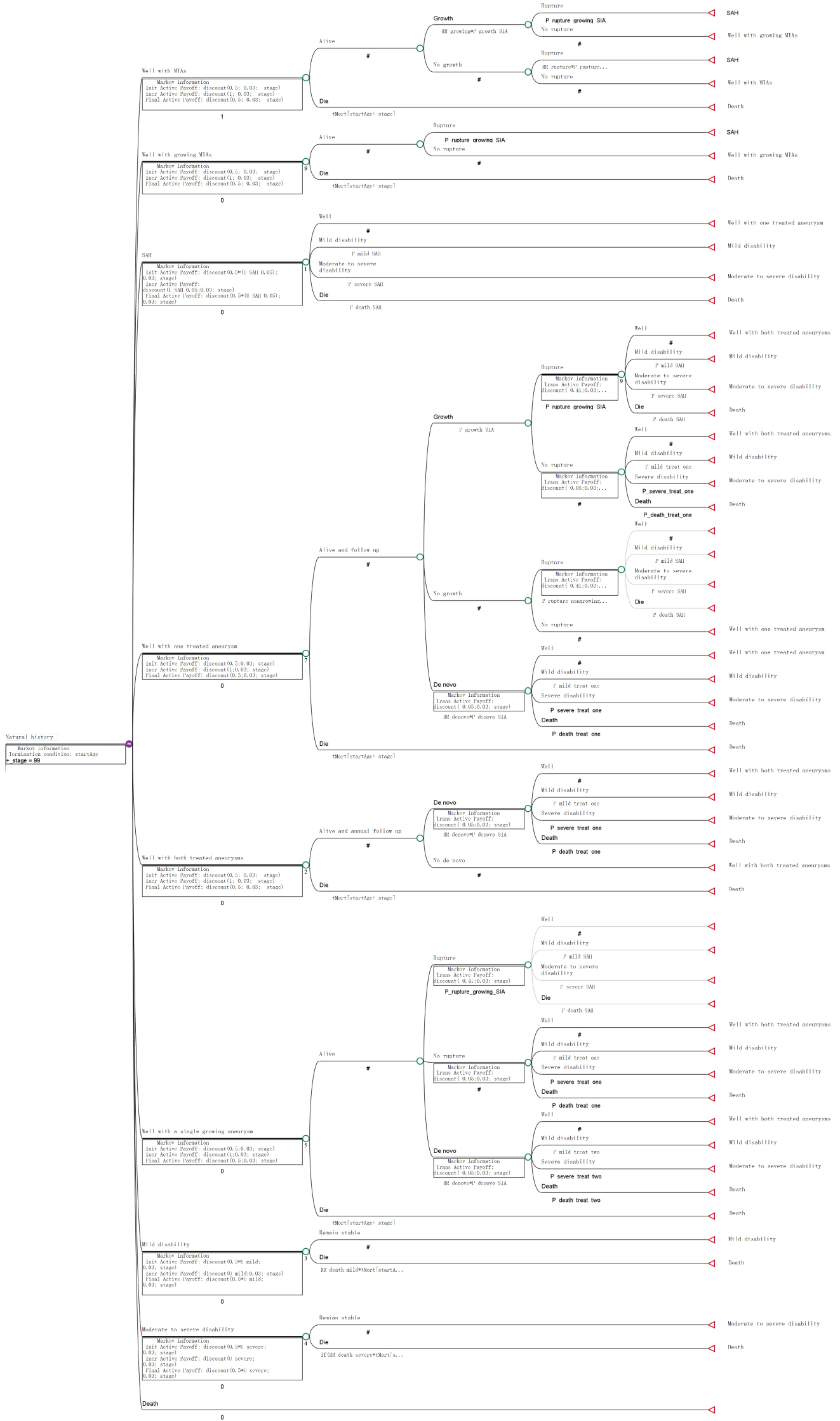

Supplement: Supplementary file 1 [file Image_1.PDF]
